# The effect of local repeated passive heating and handgrip exercise on reflex cutaneous vascular and sudomotor responses to whole-body heat stress

**DOI:** 10.1186/2046-7648-4-S1-A119

**Published:** 2015-09-14

**Authors:** David A Low, Thomas G Bailey, Danielle Turner, N Timothy Cable, Helen Jones

**Affiliations:** 1 Research Institute for Sport and Exercise Sciences, Faculty of Science, Liverpool John Moores University, UK

## Introduction

Enhancements in local cutaneous microvascular function are dependent upon elevations in both skin blood flow and temperature with repeated episodes of shear stress a key mechanism [1,2]. The mechanisms cause chronic sudomotor adaptations are not entirely clear however. Acute alterations in local cutaneous blood flow and temperature independently modify local sweat rate [3], possibly via variations in shear stress affecting endothelial nitric oxide release, suggesting that changes in skin blood flow and/or temperature may modulate sudomotor adaptations. It is also unclear as to the effects of localised thermal/hemodynamic interventions on neural cutaneous vascular and sudomotor responses. The aim of this study was to therefore compare the effects of repeated local forearm passive heating (that increases skin temperature and skin blood flow) and local handgrip exercise (that increases skin blood flow only) on sudomotor responses to whole-body heat stress.

## Methods

Fourteen healthy young males [age 21(3) y] underwent 8 weeks of local passive heating on 1 arm (submersion of the arm up to elbow in 40 °C water) and intermittent handgrip exercise (33% MVC; 30 contractions per min) training with the other (treatment arms were randomised) for 3 sessions per week for 30 minutes each in thermoneutral conditions. Whole-body passive heat stress in a water-perfused suit (~48 °C) was employed to obtain mean body temperature thresholds and sensitivities for bilateral forearm sweat rate and cutaneous vascular conductance (CVC) prior to and following the interventions.

## Results

Following both interventions mean body temperature thresholds for both forearms sweat rate [Heating; 36.94(0.28) vs. 36.88(0.18) °C, Exercise; 36.95(0.29) vs. 36.85(0.18) °C, all P > 0.05] and cutaneous vasodilation [Heating; 36.94(0.25) vs. 36.85(0.14) °C, Exercise; 36.94(0.27) vs. 36.85(0.14) °C, all P > 0.05] did not change. Similarly, both forearms sweating [Heating; 0.24(0.22) vs. 0.20(0.16) mL.cm^2^.min.°C^-1^, Exercise; 0.18(0.09) vs. 0.22(0.14) mL.cm^2^.min.°C^-1^, all P < 0.05] and skin blood flow sensitivities [Heating; 5.86(3.28) vs. 4.11(1.21) CVC units.°C ^-1^, Exercise; 5.53(3.37) vs. 4.80(1.27) CVC units.°C ^-1^, all P < 0.05] were also unchanged following both interventions.

## Discussion

Interventions, such as heat acclimation or exercise training, improve thermal sweating via a combination of increased capacity and sensitivity of sweat glands and changes in central thermoregulatory responses. Nevertheless, the actual signal(s) for sweating adaptations is unclear. Early research indicated that repeated forearm heating or electrical stimulation did not affect forearm sweat rate during whole-body passive heating, similar to the present study [3]. Sweating would not have occurred on the handgrip forearm and sweating on the heated forearm may have been blunted/impaired by water immersion in the current study. Previously observed enhancements in microvascular function with local passive heating, in contrast to the present findings, were evident during local, e.g., non-neural, heating [1].

## Conclusion

These results suggest that 1) any effects of local interventions aimed at the cutaneous vasculature do not result in changes in the neural cutaneous vascular and sudomotor responses to whole-body passive heating, and, 2) a combination of repeated elevations in internal temperature and sweating are required for sudomotor adaptations.
